# A primary effect of palmitic acid on mouse oocytes is the disruption of the structure of the endoplasmic reticulum

**DOI:** 10.1530/REP-21-0332

**Published:** 2021-12-03

**Authors:** Yisu Wang, Iestyn Pope, Henry Brennan-Craddock, Emma Poole, Wolfgang Langbein, Paola Borri, Karl Swann

**Affiliations:** 1 School of Biosciences, Cardiff University, The Sir Martin Evans Building, Museum Avenue, Cardiff, UK; 2 School of Physics and Astronomy, Cardiff University, The Parade, Cardiff, UK

## Abstract

Exposure of mouse oocytes to saturated fatty acids (FAs) such as palmitic acid (PA) has been shown to increase lipid content and cause an endoplasmic reticulum (ER) stress response and changes in the mitochondrial redox state. PA can also disrupt Ca^2+^ stores in other cell types. The links between these intracellular changes, or whether they are prevented by mono-unsaturated FAs such as oleic acid (OA), is unclear. Here, we have investigated the effects of FAs on mouse oocytes, that are maturated *in vitro*, using coherent anti-Stokes Raman scattering and two-photon fluorescence microscopy. When oocytes were matured in the presence of PA, there were changes in the aggregation pattern and size of lipid droplets that were mitigated by co-incubation in OA. Maturation in PA alone also caused a distinctive disruption of the ER structure. This effect was prevented by incubation of OA with PA. In contrast, maturation of mouse oocytes in medium containing PA was not associated with any significant change in the redox state of mitochondria or the Ca^2+^ content of intracellular stores. These data suggest that a primary effect of saturated FAs such as PA on oocytes is to disrupt the structure of the ER and this is not due to an effect on the mitochondria or Ca^2+^ stores.

## Introduction

Fatty acids (FAs) such as palmitic acid (PA) and oleic acid (OA) are two predominant saturated- and unsaturated FAs respectively in mammalian serum and follicular fluid (Dunning *et al.* 2014). PA has been commonly used to induce and study the lipotoxicity in various cell types (Ertunc & Hotamisligil 2016). The incubation of mammalian oocytes in PA has also been used to study the effects of lipotoxicity on reproduction because it can mimic many of the detrimental effects of high-fat diets on subsequent oocyte quality and embryo development (Dunning *et al.* 2014, Sutton-McDowall *et al.* 2016, Bradley & Swann 2019). High concentrations of PA in the culture medium are rapidly incorporated into the cellular lipid pool, mainly as saturated triacylglycerol (TAG), diacylglycerols (DAG) and phospholipids (PLs), thereby significantly altering the composition of the PL pool (Leamy *et al.* 2014, Shen *et al.* 2017). Oversaturation of PL membrane species is associated with stiffing the cellular membrane and leads to various problems in membrane trafficking (Rintoul *et al.* 1978, Schroeder & Goh 1980, Spector & Yorek 1985). Electron microscopy shows that chronic PA treatment causes endoplasmic reticulum (ER) dilation in hepatic cells (Leamy *et al.* 2014), pancreatic β-cells (Karaskov *et al.* 2006) and Hela cells (Shen *et al.* 2017). Drastic changes in fluidity and rigidity of the ER impair its function which disrupts protein secretion pathways and evoke adaptive mechanisms which are known as the unfolded protein response (UPR) (Malhotra & Kaufman 2007*a*). The UPR activates a series of compensatory reactions via three major branches of signalling pathways and the activation of ER-associated protein degradation (Kaufman 1999, Han & Kaufman 2016). If membrane fluidity is not normalized, prolonged ER stress leads to the stimulation of cell death pathways that are either dependent or independent of mitochondria (Malhotra & Kaufman 2007*b*, Vannuvel *et al.* 2013).

High-fat diets have a detrimental effect on reproduction in mammals, and most of these effects are mediated via damage caused to the immature oocyte or ovulated egg. Many of the effects of a high-fat diet can be mimicked by the *in vitro* culture of mammalian oocytes in saturated fats, such as PA, for relatively short periods of about 10–24 h (Barnett & Bavister 1996). The incubation of oocytes in saturated PA and other FAs also mimics the exposure of animals to high-fat diets by leading to an increase in the overall lipid droplet (LD) content (Dunning*et al.* 2014, Carta *et al.* 2017). It has been shown that high PA treatments were able to induce the same UPR and exhibit markers of ER stress in both *in vivo* and *in vitro* mouse oocyte models (Wu *et al.* 2010, 2012). PA treatment has also been shown to induce ER stress during* in vitro* maturation (IVM) of cattle oocytes and cumulus cells (Sutton-McDowall *et al.* 2016). It is not clear exactly how either high-fat diets, or the incubation of maturing oocytes *in vitro* in saturated FAs, alter the ER and lower their quality. It is notable that PA-induced ER stress is mimicked by agents that cause efflux of Ca^2+^ from the ER to the mitochondria. PA could affect other organelles such as mitochondria since the release of Ca^2+^ could lead to mitochondrial uptake and enhanced reactive oxygen species (ROS) production (Wu *et al.* 2010, 2012, Bradley & Swann 2019). It has been reported that oocytes from mothers fed a high-fat diet have abnormal mitochondrial morphology, impaired electron transport and redox state (FADH or NADH levels). However, is not clear whether PA directly affects Ca^2+^ homeostasis or the mitochondrial activity.

In contrast to saturated FAs, unsaturated FAs such as OA can have positive effects on oocytes development, promoting maturation and even in reversing some of the negative effects caused by saturated FA (Aardema *et al.* 2011). OA is the second most abundant FA after PA in high-quality oocytes whereas stearic acid is the second most abundant FA in low-quality oocytes (Kim *et al.* 2001). This suggests that the FA composition of oocytes influences development competence. In somatic cells, the co-treatment with OA and PA was found to suppress dysfunction and dilation of the ER membrane, reduce PA incorporation into PL pool and restore overall membrane saturation (Leamy *et al.* 2014). OA may be able to prevent PA-induced ER stress and cell death by increasing the conversion of PA into triglyceride (Listenberger *et al.* 2003, Borradaile *et al.* 2006, Aardema *et al.* 2013). However, the effects of OA and PA on ER and LD content structure have not been characterized in mouse oocytes.

We have previously shown that LD number and spatial distribution can be assessed in a quantitative, chemically specific, non-invasive way in live mouse oocytes and mature eggs using label-free coherent anti-Stokes Raman scattering (CARS) microscopy (Bradley *et al.* 2016). In this paper, we use quantitative CARS imaging of *in vitro* cultured mouse oocytes to investigate how PA and OA treatments affect the mitochondria, Ca^2+^ homeostasis and cytoplasmic morphology. We show that high concentrations of PA lead to a substantial change in the size distribution of LDs in mouse oocytes and yet have no consistent effect upon the mitochondrial redox status or intracellular Ca^2+^ store content. Instead, our data suggest that PA-induced lipotoxicity is associated with the oversaturation of the ER membrane which leads to the formation of abnormal sheet structures. OA was able to reverse the detrimental effects of PA and was associated with the sequestration of exogenous PA into LDs.

## Materials and methods

### Gamete collection and culture

Germinal vesicle (GV) stage oocytes forIVM were collected from the punctured ovarian follicles of female CD1 mice (8–10 weeks old) that had been culled by cervical dislocation. All animals were handled according to UK Home Office regulations, and procedures were carried out under a UK Home Office Project License with the approval of Cardiff University Animal Ethics Committee. Cumulus cells were removed by gentle pipetting (for GV oocytes). For all experiments, oocytes were split and some were kept in normal M2 medium (Sigma) at 37°C for 15 h as the control as used previously in studies of maturation (Yu *et al.* 2010). The other half were kept in drops of M2 medium supplemented with either 200 μM PA (Nu-Chek Prep Inc., Elysian, MN, USA), 200 μM OA (Sigma) or combination of 200 μM PA and 200 μM OA at 37°C for 15 h. Live imaging experiments were carried out with oocytes or mature eggs cultured in HKSOM as described previously. All drops were covered with mineral oil (embryo tested, Sigma) to prevent evaporation.

The FAs were prepared using a two-step protocol that involved preparing a BSA solution and a PA solution, and subsequently adding the PA to the BSA for the final conjugation. In the first step, 95 mg FA-free BSA was dissolved in 9.5 mL of PBS and kept in a 55–60°C water bath. Then, 12.8 mg of PA was dissolved in 0.5 mL of 0.1 M NaOH at 55–60°C. After vortex mixing, the suspension was heated in a boiling water bath until the solid PA was dissolved. In the second step, 0.5 mL of the 0.1 M PA solution was poured into the 9.5 mL of the BSA–PBS solution while both the solutions were hot. The final solution was then kept warm in a 55–60°C water bath for at least 15 min until the solution became clear. The FA conjugated BSA solution was stored at −20°C, and the stock solutions were warmed up in a 55–60°C water bath before dilution on the day of use. The same procedure was used for conjugating OA to BSA.

### CARS, TPF and DIC microscopy

An in-house-built imaging dish was used, consisting of a 25-mm diameter glass coverslip and a removable glass lid. Petroleum jelly was spread around the coverslip before it was held by a silicon gasket for an airtight seal. A drop of M2 medium (800 μL) was placed in the centre of the glass coverslip and covered with mineral oil. Live oocytes were then pipetted into the drop, and the whole sample dish was maintained at 37°C on the microscope stage. All the images were acquired with a 60× 1.27NA water immersion objective (Nikon λS series) and a 0.72 NA dry condenser. The CARS microscope was set up as described in (Pope *et al.* 2013), with two-photon fluorescence (TPF) and differential interference contrast (DIC) imaging capabilities. Three-dimensional CARS images were taken as z-stacks over 50 μm depth in 0.5 μm steps. CARS in-plane xy images were taken with 0.1 μm pixel size, typically in an 80 μm × 80 μm frame, with 0.01 ms pixel dwell time, and time-average total power of ~20 mW at the sample.

TPF imaging was performed on ER stains absorbing at 484 nm and emitting at 501 nm, simultaneously with CARS. For this purpose, we use a third near-infrared beam centred around 930 nm, hence, separated from the CARS excitation (pump and Stokes) beams and independently optimized in time domain to provide Fourier-limited pulses of ~30 fs duration at the sample for maximum TPF excitation. TPF was detected simultaneously with CARS in the wavelength range 498–540 nm via appropriate dichroic beamsplitters and band pass filters (Semrock, Rochester, New York, USA) in front of a second photomultiplier (Hamamatsu, H10770A-40).

Hyperspectral images were acquired by changing the relative delay time between pump and Stokes pulses which are equally linearly chirped, resulting in a tuning range of the instantaneous frequency difference across the 1200–3800 cm^−1^ vibrational range (Pope *et al.* 2013). In the present work, scans were taken over the range 2200–2400 cm^−1^ for deuterated lipids and over the range 2400–4400 cm^−1^ for non-deuterated samples, in 5 cm^−1^ steps over an area of about 10 μm × 90 μm (xy region), with 0.1 μm pixel size and a pixel dwell time of 0.01 ms. An in-house developed software (Masia *et al.* 2015) was used to retrieve Raman-like spectra from CARS spectra, as summarized here. First, hyperspectral images were background-corrected by subtracting an image measured under identical excitation and/or detection conditions but with pump and Stokes pulses out of time overlap. They were then noise-filtered using singular value decomposition on the square root of the CARS intensity, retaining only components above noise. CARS intensity ratios were calculated by dividing the background-corrected CARS intensity by the corresponding CARS intensity measured in water under the same excitation and detection conditions. The phase-corrected Kramers–Kronig method was used to retrieve the complex CARS third-order susceptibility from the CARS intensity ratio (normalized to the non-resonant value in glass using a known water–glass ratio), which is linear in the concentration of chemical components (Masia *et al.* 2013). Shown spectra are the retrieved imaginary part of the susceptibility (Im(χ)) normalized to the total area, and Origin software (OriginLab) was used to plot these against oocytes or embryos of the same developmental stage and/or conditions.

All the DIC images were acquired by a monochrome ORCA-285 Hamamatsu CCD camera with 120 ms frame exposure time and a 12.5° polarisation angle in the de-Senarmont DIC illuminator (yielding a 25° phase offset). A motorized stage and z-drive (Prior ProScan III) enabled lateral xy sample movement and axial z focus movement for focusing. MicroManager software (Edelstein *et al.* 2010) was used for automated z-stacks acquisition over the full cell-depth (~70 μm) in 1 μm steps.

### LD aggregate analysis

As described in Bradley *et al.* (2016, 2019), single-frequency CARS images were fitted in 3D by a in-house-developed software (CCDPlot), to obtain the 3D position coordinates, x, y, z widths and amplitude maxima of LDs from the fit. An Origin script was used to define and count LD clusters. More specifically, an index was assigned to LDs spatially separated from their nearest neighbour by less than a given value L (equal to 1.5 times the distance in resolution units), with the index being identical for all LDs belonging to the same cluster (Bradley *et al.* 2016, 2019). The occurrence of this index was then used to calculate the number of LDs in each aggregate (called aggregate size) and the number of aggregates. The probability P_k_ of each size k = 1, 2, 3, etc. (including 1, i.e. isolated LDs) was calculated from the histogram of the aggregate size by using P_k_=Ok/N, where N is the total number of LDs and O_k_ is the occurrence of size k. For each oocyte, the mean square size was calculated as <s^2^> =Sum(k-1)P_k_ where 1 is subtracted to obtain the number of partner LDs in an aggregate. The extent of LD aggregation was calculated as the square root of the mean square aggregate size (sqrt(<s^2^>)). To compare populations of eggs across different IVM conditions, the average sqrt(<s^2^>) per population of eggs at each condition is plotted (OriginLab) against the population-averaged total number of LDs at that condition.

### Analysis of LD volume

As described in Bradley *et al.* (2016, 2019), for each LD, its volume V_i_ in μm^3^ is calculated by multiplying the LD widths in x, y, z (obtained from the CCDPlot analysis). To account for sub-resolution droplets (which manifest as having a lower CARS amplitude and a resolution-limited width), the volume of each LD was normalized as V_in_ = V_i_(A_i_/A_b_), where A_i_ is the CARS amplitude (square root of the CARS intensity) of the droplet and A_b_ represents the amplitude of the brightest droplet measured in the oocyte (assumed to be not resolution limited). The probability (P_Vh_) of LD to fall within a given volume range V_h_ was calculated from the histogram of V_h_ as P_Vh_ = O_Vh_/N where O_Vh_ is the occurrence of V_h_, and N is the total number of LDs. The mean volume was then calculated as 
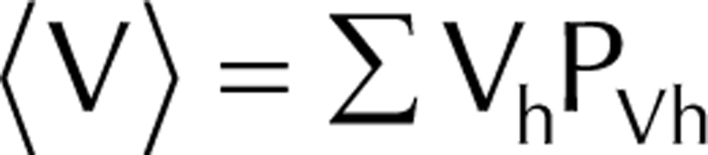
 for each egg. To compare different experimental conditions, the average of 〈V〉 was calculated per population of eggs at each condition and plotted (OriginLab) against the population-averaged total number of LDs at that condition.

### FAD autofluorescence assay of redox potential

Epifluorescence measurements of FAD autofluorescence were performed using a Nikon Ti-U microscope with a 20× objective. Excitation light was provided by a light-emitting diode (Cairn Research Ltd, Faversham, Kent, UK), using a 460-nm excitation filter (10 nm BP), and emission light was imaged with a Photometrics CoolSnap HQ2 CCD camera through a 510–550 nm-emission filter alongside a 505-nm dichroic mirror. The zona pellucida of the oocytes was gently removed by acid Tyrode’s solution (Sigma). The oocytes were then transferred to a glass-bottomed dish filled with 0.9 mL HKSOM medium (covered in mineral oil to prevent evaporation) and kept at 36^o^C throughout the experiment. Drugs were dissolved in HKSOM medium and added to the sample dish by pipetting in a 100 mL volume of a solution that was 10× the required final concentration. ImageJ with the MultiMeasure Plug-in was used to collect data per oocyte, when selecting regions of interest. The fluorescence intensities from the image data were normalized by: 
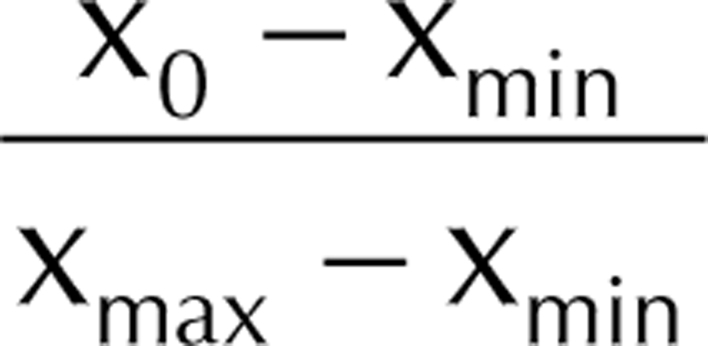
, where x_0_ is the resting fluorescence level before drug addition and x_min_ and x_max_ are the minimum and maximum fluorescence levels induced by cyano-4 hydroxycinnamate (CN; Sigma) and carbonyl cyanide-4-(trifluoromethoxy)phenylhydrazone (FCCP; Sigma), respectively. This allowed the calculation of the contribution of β-oxidation to the redox potential. The intensity of fluorescence emission is presented in arbitrary units.

### Luminescence assays of ATP

Whole-cell luminescence assays were performed by transferring individual whole oocytes into 200 μL CellTiter-Glo® Luminescent Cell Viability Assay reagent (Promega). The luminescence signal was measured for 5 min in a custom-made luminometer with a cooled S20 photomultiplier tube in photon counting mode (ET Enterprises Ltd., Uxbridge, UK). The data were collected using the ET Enterprises (formerly Electron Tubes) RS232 Photon Counting software. The steady state level of luminescence was taken as a measure of ATP per oocyte.

### Measurements and analysis of intracellular Ca^2+^

Meiotic metaphase II (MII) oocytes were microinjected with Ca^2+^-sensitive fluorescent dye Oregon Green 488 BAPTA-1 dextran (OGBD, 494/523 nm) and the non-Ca^2+^-sensitive fluorescent dye Alexa Fluor Dextran 594 (Alexa, 590/617 nm) in a ratio of 10:1 which was then diluted in a KCl HEPES buffer (120 mM KCl, 20 mM HEPES at pH 7.4). Injections were performed within a shallow drop of M2 medium (covered with mineral oil to avoid evaporation) on a microscope stage (Nikon TE2000). A full description of the method is given elsewhere (FitzHarris *et al.* 2018). To introduce a small amount of Ca^2+^ dye to the oocyte (~3–5% its volume), a Picopump (WPI Ltd, Hitchin, Herts, UK) was used to provide a short pressure pulse of ~20 psi. The zona pellucida were removed from the oocytes using acid Tyrode’s treatment prior to imaging. Oocytes were then transferred onto a glass-bottomed dish filled with 900 mL HKSOM Ca^2+^-free solution (covered in mineral oil) and kept at 36^o^C through the experiment. Drugs were dissolved in HKSOM medium and added to the sample dish by pipetting. Epifluorescence measurements were performed using a Nikon Ti-U microscope with a 20× objective. Excitation light was provided by a halogen lamp, and emission light was collected by a Photometrics CoolSnap Q2 CCD camera through 505 nm dichroic mirror. Two sets of excitation/emission filters were controlled by the software for the two fluorescent dyes: 490 nm bandpass (BP) filter/510–550 nm BP filter for OGBD, and 550 nm BP filter/600 nm-long pass filter for Alexa 2013. The fluorescent signals of both dyes were measured using the time-lapse imaging mode of Micromanager software (https://micromanager.org/) where an image was captured every 10 s. The oocytes were first imaged for 10 min to measure the baseline fluorescence. Then, 10 μM thapsigargin and 5 μM ionomycin were sequentially added to the sample dish to induce Ca^2+^ release from the intracellular store. To analyse the cytosolic Ca^2+^ changes, the fluorescence signal from OGBD was divided by the signal from Alexa in each oocyte. This normalized the Ca^2+^ response variation between oocytes and avoided difference due to the amount of dye injected.

### Statistical analysis

For the Ca^2+^ store and FAD autofluorescence experiments, each data group (with eggs from the same mice) was tested for a normal distribution in R using the Shapiro–Wilk test. If the group was normally distributed (*P* > 0.05) then a Student’s *t*-test for difference in means was performed between the control group and treated group, using excel. If the Shapiro–Wilk value (*P* < 0.05) indicated that the data group was not normally distributed. Data significance was tested using a Wilcoxon signed-rank test. F test was carried out in R on each data set to compare the variance of the data. If the variance was equal, a homoscedastic *t*-test was performed, and if the variance was unequal, a heteroscedastic *t*-test was performed.

## Results

### PA causes changes in the size and distribution of LDs in mouse oocytes

**Figure 1:**
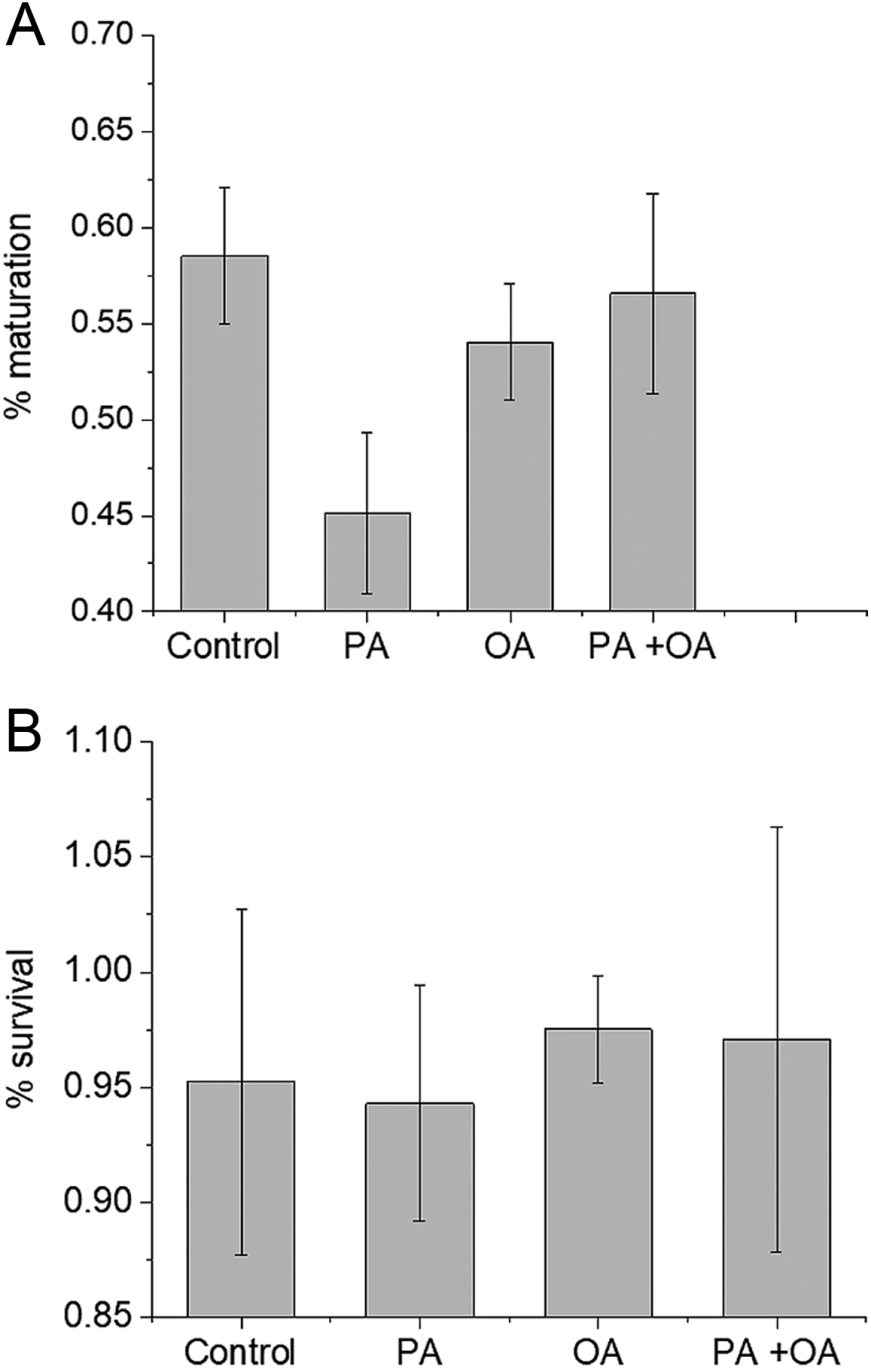
Oocyte maturation and survival during IVM in the presence of FAs. Oocytes treated with 200 μM PA (*n* = 269) showed a significant reduction (*P* < 0.05) from controls (*n* = 324) in their ability to mature to the MII stage. Oocytes matured in 200 μM OA (*n* = 223) or 200 μM PA + 200 μM OA (*n* = 23) showed no significant reduction or gain in maturation success from controls during IVM (*P* > 0.2). (A). After 15-h incubation period for IVM, maturation to MII was recognized by the presence of polar body. These cells, together with live immature oocytes (with clear GV), were classed as survived eggs with no significant difference between groups (all *P* > 0.2) (B). The error bars represent the s.d.

We investigated the effect of FAs on mouse oocytes by incubating immature (GV stage) oocytes in medium containing albumin-conjugated PA, or OA or both for 18 h to allow maturation to MII stage. *In vitro* culture of mouse oocytes usually leads to the disappearance of the GVs (after ~ 2 h) and maturation to the MII stage after about 15–18 h. We found that oocytes treated with 200 μM PA showed a significant decrease in maturation success (percentage of M2 eggs with a clear first polar body in the sample population) in comparison with the control oocytes (Fig. 1). Incubations in higher concentrations of PA (e.g. 400 μM) lead to lysis of many oocytes (unpublished observations). These data confirm previous studies showing that PA has deleterious effects on the developmental competence of GV oocytes (Dunning *et al.* 2014). In contrast to PA, a concentration of 200-μM OA had no significant impact on oocytes survival or maturation rate. Furthermore, the combination of 200 μM OA and 200 μM PA in the culture medium also had no significant effect on mouse oocyte survival or maturation compared to control oocytes, suggesting that OA offsets the deleterious effects of PA. These data show that *in vitro* culture of mouse oocytes provides a simple system to investigate the deleterious effects of PA on maturation and how unsaturated FAs such as OA may mitigate some of the effects of saturated FAs.

**Figure 2:**
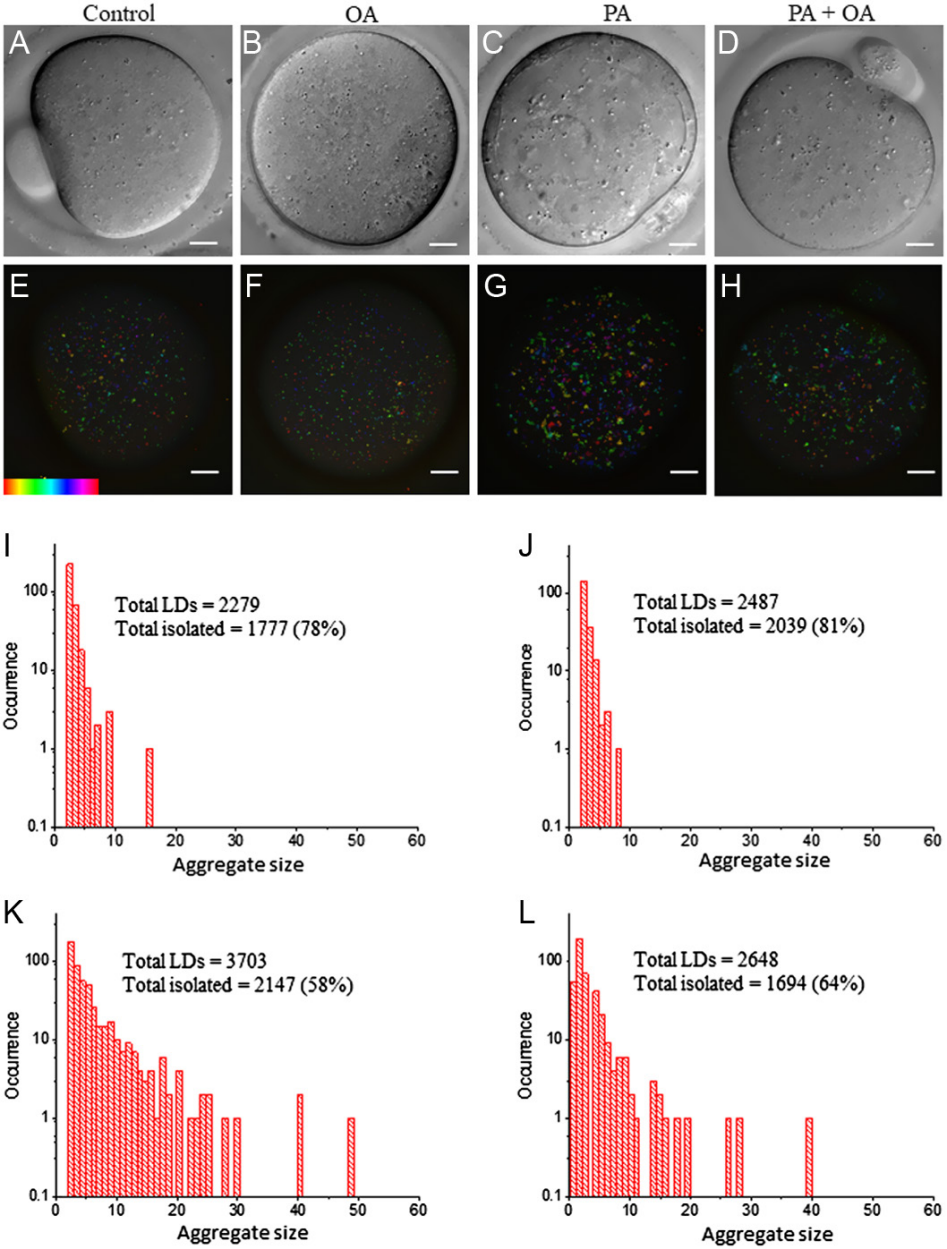
CARS images of LDs in live MII oocytes matured in high-FA environments. Immature GV stage oocytes were incubated overnight in standard M2 medium (A, E, I) supplemented with 200 µM PA (B, F, J), 200 µM OA (C, G, K) or a combination of 200 µM PA and 200 µM OA (D, H, L). MII oocytes were then selected for DIC (A, B, C, D) and CARS imaging (E, F, G, H). CARS images are colour-coded; colour bar shows depth colour-coding from −25 to 25 μm of 101 z-stacks (0 μm being the approximately equatorial plane of the egg or embryo). (I, J, K, L) are representative histograms of the number of LDs making up clusters for the different growth conditions above.

We have previously shown that CARS microscopy provides label-free, chemically specific, quantitative 3D imaging of LDs in live mouse oocytes. Here, we used single-frequency CARS imaging of LDs at the CH_2_ symmetric stretch vibration (2850 cm^−1^) which is the abundant acyl chain of both PA and OA. Figure 2E shows a typical CARS image of LDs in MII oocytes that had matured in M2 medium for 18 h. As reported previously, LDs are present throughout the oocyte with some LDs forming clusters (Fig. 2E and I). LDs in MII oocytes that had been matured in M2 medium containing 200 μM PA showed an increase in the total number of LDs (Fig. 2F and J). In addition, LDs had formed into large clusters throughout the cytoplasm. Some of these clusters were also visible in the DIC images (Fig. 2B compared to A). In contrast, MII oocytes that had been matured in 200 μM OA had a wide spatial dispersion of LDs throughout the cytoplasm and had a similar number of LDs to that seen with control oocytes (Fig. 2G and K). The relative lack of LDs in OA-treated oocytes was also evident from the lack of clusters seen in the DIC image (Fig. 2C). There was an increase over controls in the number of LDs in MII oocytes with the PA and OA co-treatment (Fig. 2D, H and L). However, they formed less clusters than PA-treated oocytes (Fig. 2L), suggesting that OA reduces some of the aggregating effects of PA.

**Figure 3:**
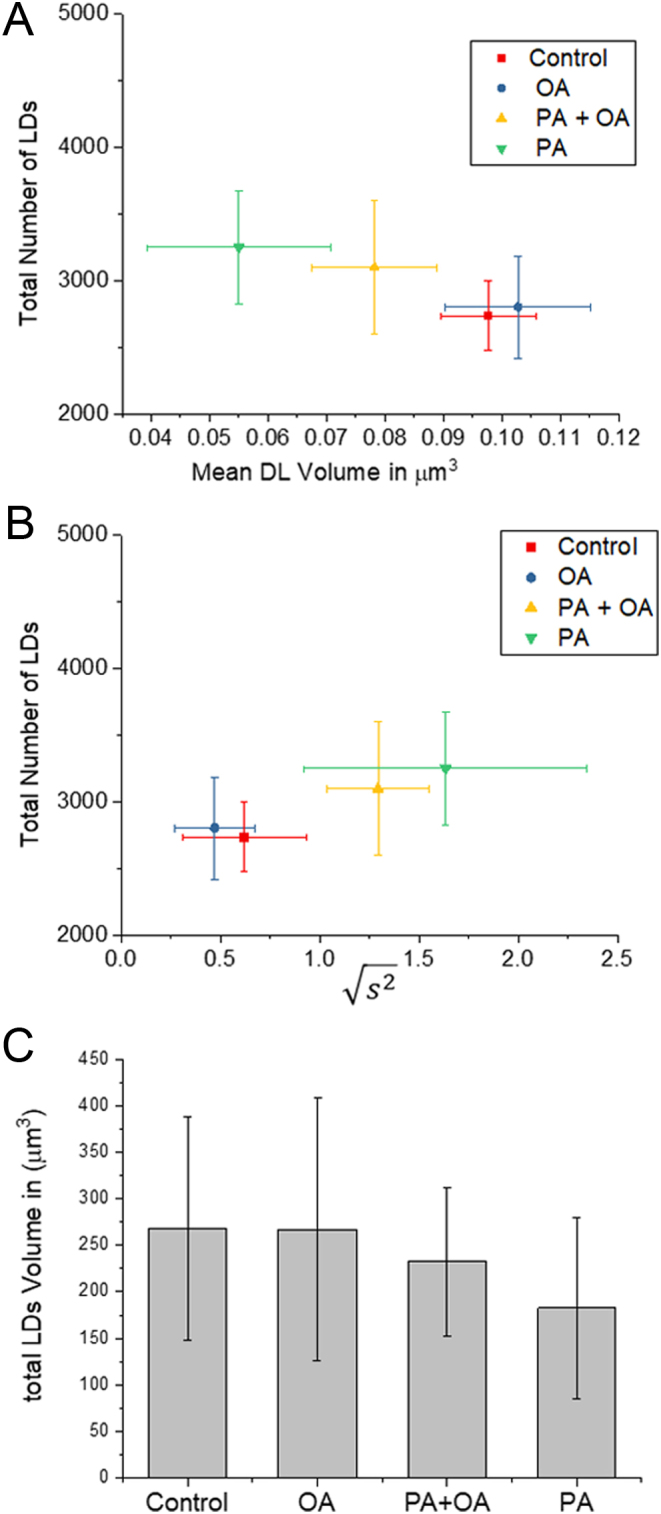
LD contents and aggregation size with FA treatments. (A) Scatter plot of the square root of the mean squared aggregate size 
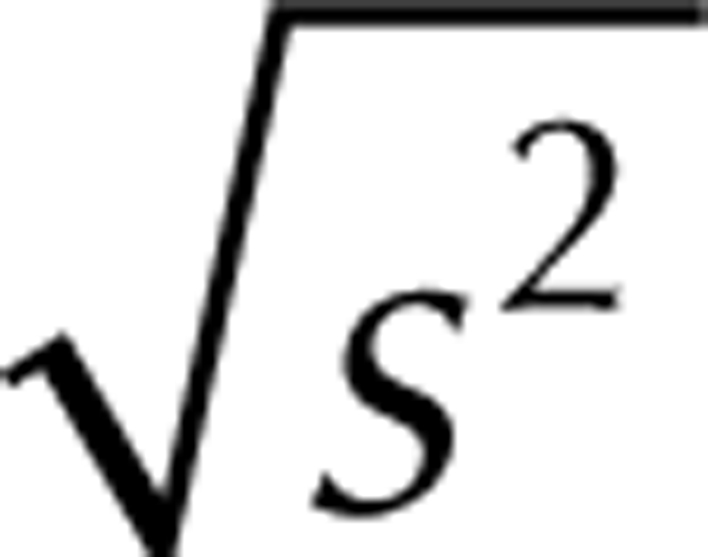
 against the total number of LDs in ensembles of MII oocytes imaged immediately after overnight incubation in standard M2 medium (red square, *n* = 12), or supplemented with 200 µM PA (green triangle, *n* = 10), 200 µM OA (blue circle, *n* = 8) or combination of 200 µM PA and 200 µM OA (yellow triangle, *n* = 8). The distribution of each variable in the corresponding ensemble is shown as the average ± s.d. (B) LD volume *V* in μm^3^ against total number of LDs. The distribution of each variable in the corresponding ensemble is shown as the average ± s.d. All data represent multiple trials, using two to three mice per trial. (C) The total lipid content of oocytes with the three different treatments calculated as the product of the volume and total number of LDs.

Quantitative analysis of the aggregation patterns within the oocyte was performed as described by Bradley *et al.* (2016). The occurrence of aggregate sizes is presented in Fig. 3. The volume of LDs (see ‘Materials and methods’ section) was calculated as a population average for all oocytes under each condition (Fig. 3A). In comparison with controls, LDs in PA-treated oocytes were slightly smaller (*P* < 0.01), while LDs in OA-treated oocytes were similar in size. LDs treated with a combination of PA and OA had a size that was larger than PA-treated oocytes (*P* < 0.01), but smaller than OA-treated oocytes (*P* < 0.05). The LD aggregate size (see ‘Materials and methods’ section), as an average per population of oocytes in each different condition, is also plotted against the total number of LDs (Fig. 3B). In comparison with controls, the aggregate size of LDs with PA is increased (*P* < 0.01), whereas OA did not induce any clustering effect. Furthermore, OA mitigates the PA-induced clustering because oocytes treated with OA plus PA had a smaller aggregate size (*P* < 0.01) compared to oocytes treated with PA alone (Fig. 3B). All the FA-treated oocytes had on average a slightly increased number of LDs compared to controls. Overall, the LD number of sizes suggest that the total LD lipid content (the product of LD number times size shown in Fig. 3C) is reduced in PA-treated oocytes compared to all other cases (*P* < 0.01). These data suggest that the main effect of PA is on the pattern of distribution of LDs in oocytes.

### PA does not alter mitochondrial redox state or ATP levels in mouse oocytes.

**Figure 4:**
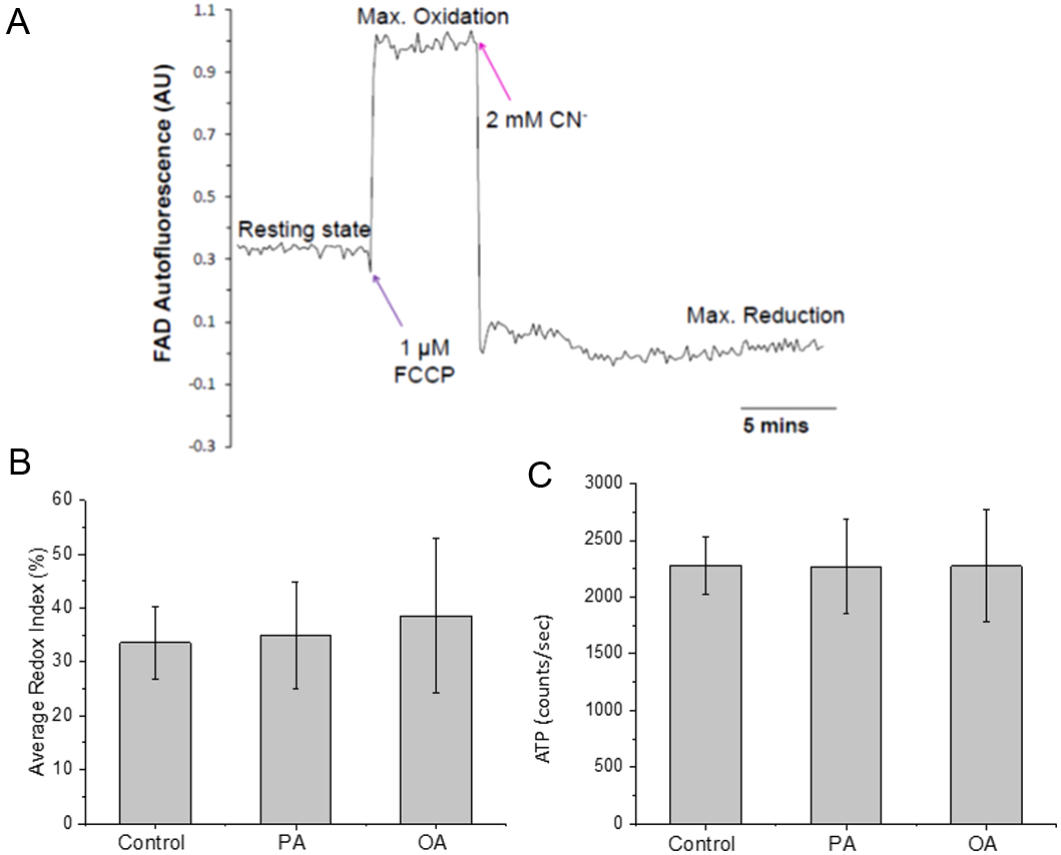
Mitochondrial metabolism of living MII oocytes matured in high-FA environments. An example of traces of mitochondrial FAD autofluorescence signal in MII oocytes subjected to induction of maximum reduction (2 mM cyanide) followed by subsequent maximum oxidation (1 μM FCCP). (B) The average FAD redox indexes of MII oocytes matured with various FA condition. The FAD redox index of each oocyte is the resting fluorescence level before adding drugs normalized by fluorescence levels of the maximum reductions state and maximum oxidation state. Data represent multiple oocytes (*n* = 50, 34 or 26 for control, PA or OA condition, respectively) using two or three mice trials. Both PA and OA condition showed no significant difference (*P* > 0.05) from controls. (C) The luminescence of individual oocyte from each condition was measured separately after IVM; luminescence levels are shown as counts per second, and the average ATP level of each condition was calculated from multiple oocytes (*n* = 20, 24 or 22 for control PA or OA condition, respectively), using two or three mice trials. Both PA and OA condition showed no significant difference (*P* > 0.5) from controls.

We have previously demonstrated a link between the LD spatial distribution in MII oocytes and β-oxidation. Therefore, we examined if LD distribution observed in Fig. 2 is associated with FA-induced alteration in mitochondrial metabolism. The autofluorescence of the flavin adenine dinucleotide (FAD) can be used to monitor mitochondrial redox state in mitochondria which can be expressed as the relative FAD:FADH_2_ ratio. Figure 4A shows how the maximum oxidation state and maximum reduction state of FAD was established by sequential addition of the uncoupler FCCP and the cytochrome oxidase inhibitor cyanide (CN^-^). The resting redox state is then estimated from the relationship between the initial state and these two maximum and minimum values. Figure 4B shows the FAD redox state calculated for five groups of oocytes treated with different FAs. We found that there was no significant difference in the resting redox state of oocytes treated with PA or OA compared to controls. We also found no evidence for a change in FAD redox state when either PA for OA were added to immature oocytes in that way they would have been initially treated in our experimental incubation (Supplementary Fig. 1, see section on supplementary materials given at the end of this article). These data suggest that high concentrations of FAs do not have any consistent effect upon mitochondrial redox state in mouse oocytes under than conditions of our experiments.

Mitochondrial activity can also be assessed by ATP levels because isolated and maturating mouse oocytes produce nearly all of their ATP from oxidative phosphorylation (Bradley & Swann 2019). We examined the ATP content of individual oocytes that were matured with high concentration of PA or OA using a standard luciferase-based assay. The data in Fig. 4C show that treatment of oocytes for 18 h with 200 mM PA or OA did not cause any significant change in the intracellular ATP level compared to control, untreated oocytes. Together with the findings on FAD redox state, these data suggest that high-FA treatments during IVM have no clear and consistent effect on mitochondrial metabolism in mouse oocytes.

### PA does not alter intracellular Ca^2+^ homeostasis in mouse oocytes

**Figure 5:**
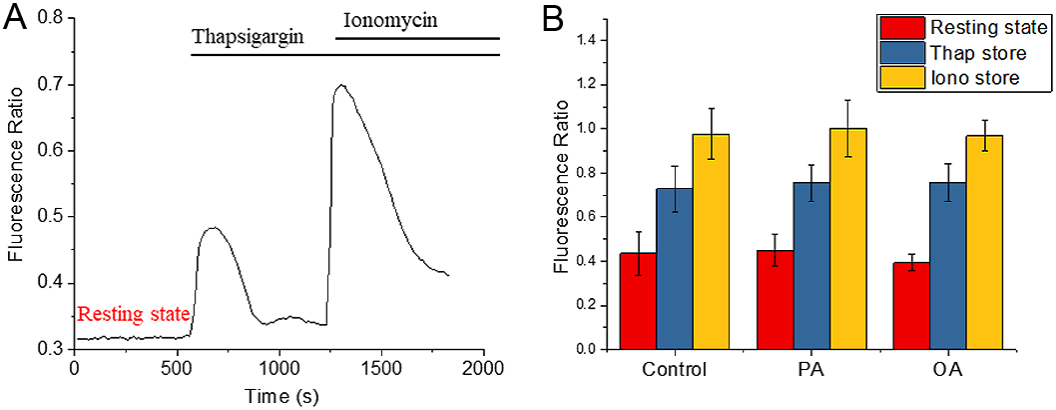
Intracellular Ca^2+^ response of living MII oocytes matured in high-FA environments. (A) After IVM, MII oocytes were injected with a mixture of OGBD and Alexa594 (in 10:1 ratio) for ratio-metric fluorescent measurements of intracellular Ca^2+^ store. The ER Ca^2+^ store was measured by the thapsigargin (10 μM)-induced Ca^2+^ response, and all other intracellular Ca^2+^ store was measured by the ionomycin (5 μM)-induced Ca^2+^ response. (B) The experiment in (A) was carried out on MII oocytes matured with various FA condition. The average fluorescence ratio of the resting state (red bar), thapsigargin response (blue bar) and Ionomycin response (yellow bar) was calculated form multiple oocytes (*n* = 55, 27 or 24 for control, PA or OA condition, respectively), using two or three mice trials. For the resting state, both PA and OA condition showed no significant difference (*P*  > 0.1) from controls. There were also no differences for the thapsigargin response (*P* > 0.5) and the Ionomycin response (*P*  > 0.1) from controls.

During IVM of a mouse oocyte, Ca^2+^ store depletion with thapsigargin can mimic high-PA treatment in causing an ER stress response (Wu *et al.* 2012). Therefore, we tested the ER Ca^2+^ store content to check for possible links with maturation in FAs. A ratio-metric dye (see ‘Materials and methods’ section) was used to normalize the Ca^2+^ response variation between oocytes as well as variations in the amount of dye injected to allow quantitative analysis. The example traces in Fig. 5A showed that ER Ca^2+^ channel blocker thapsigargin was able to induce a transient Ca^2+^ responses as the result of depletion of the Ca^2+^ store in the ER. The Ca^2+^ ionophore ionomycin invariably induced a larger response than thapsigargin as ionophores cause Ca^2+^ release from all intracellular stores. The same experiment was repeated on oocytes matured with PA or OA. The result suggests that there is no significant difference in the intracellular Ca^2+^ content between the oocytes matured in high concentrations of PA or OA compared to control oocytes. We also found that neither PA nor OA had any transient effects on Ca^2+^ levels in immature oocytes (Supplementary Fig. 1). These data suggest that the effect of PA on the LD spatial pattern in mouse oocytes is not accompanied by significant changes in Ca^2+^ homeostasis.

### PA accumulates in sheet-like structures in the endoplasmic reticulum

**Figure 6:**
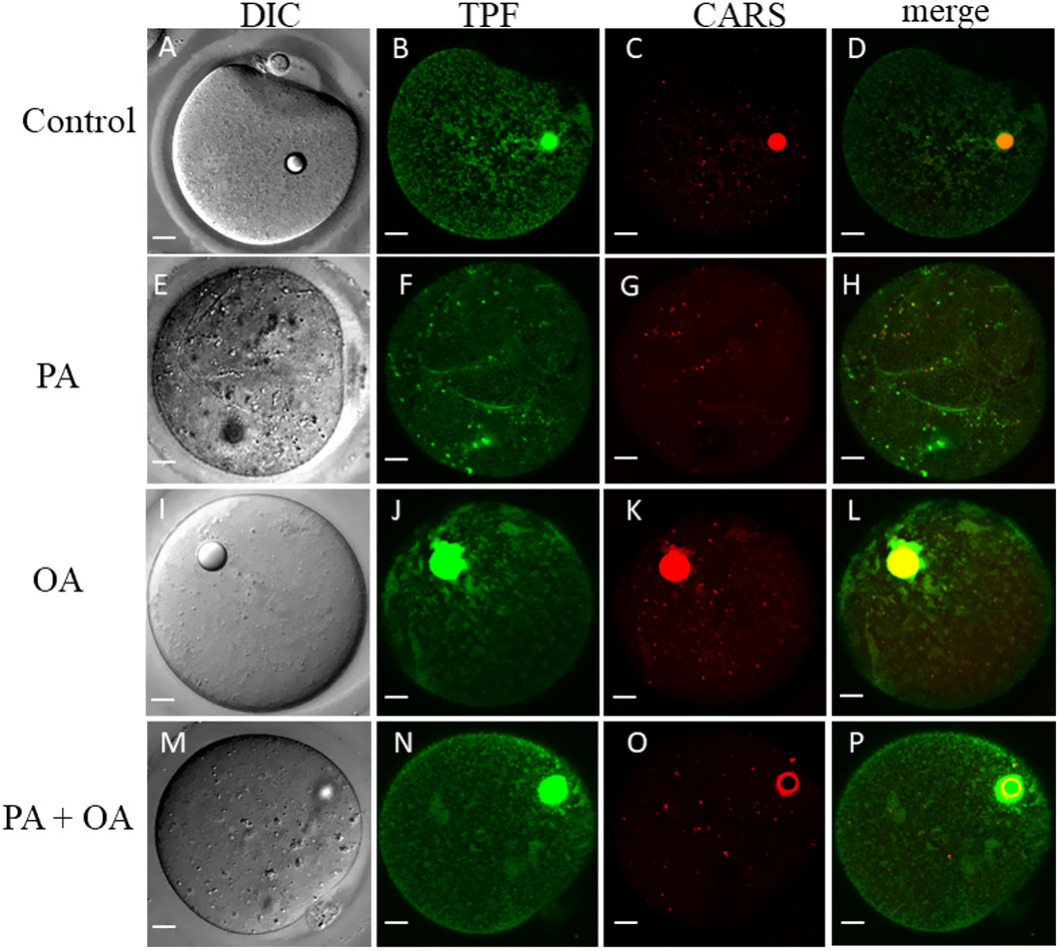
CARS, TPF and DIC images in living MII oocytes matured in different environments. For IVM, immature GV stage oocytes were incubated overnight in standard M2 medium (A, B, C, D) or supplemented with 200 µM PA (E, F, G, H), 200 µM OA (I, J, K, L) or combination of 200 µM PA and 200 µM OA (P, Q, R, S). MII oocytes were then selected and injected with neuro-DiO in a soybean oil droplet. (A, E, I and M) DIC, (C, G, K and O) false-coloured CARS images at wavenumber 2850 cm^−1^ and (B, F, J and N) TPF xy images accompanied by (D, H, L and P) false-coloured overlays scale bars: 10 μm. The bright circles inside the oocytes are due to the oil drop that was injected to load neuro-DiO into the ER.

Since PA and OA can accumulate and can be converted to PLs in the ER, we investigated the effects of FAs on intracellular membranes. The ER membrane was labelled by microinjection of an oil drop containing neuro-DiO which is a green fluorescent version of a dye previously used to label the ER in MII mouse oocytes (FitzHarris *et al.* 2003). The mature MII oocytes were then imaged using CARS and TPF microscopy simultaneously to image both the LD and the ER in the same eggs. Figure 6 and Supplementary Figure 2 show neuro-DiO labelling the ER membrane through the whole oocyte. The ER in control oocytes have distinguishable tubular structures (Nixon-Abell *et al.* 2016) with a reticular organization (Fig. 6B). In contrast, the majority of MII oocytes that had been matured in 200 µM PA developed large continuous sheet structures that were visible on TPF images (Fig. 6F). Our quantitative hyperspectral CARS analysis (Supplementary Fig. 3) shows that the sheet structures have less lipid and more water than LDs for the same sampled volume size by CARS. The neuro-DiO can only diffuse within a membrane because of its large hydrophobic headgroup. Therefore, we suggest that the sheet structures consist of layers of membranes. In some PA-treated oocytes, there are large holes (dark regions devoid of ER) on the TPF images that were not seen in the CARS image at 2850 cm^−1^ or the DIC image (Supplementary Fig. 2). Interestingly, the TPF image stacks (Supplementary Fig. 2) showed that most of the holes were, at least partially, bounded by the sheet structure, though not all the sheet structures were associated with holes. The presence of the sheet structures suggests that the PA had caused a major disruption of the ER membrane structure.

**Figure 7:**
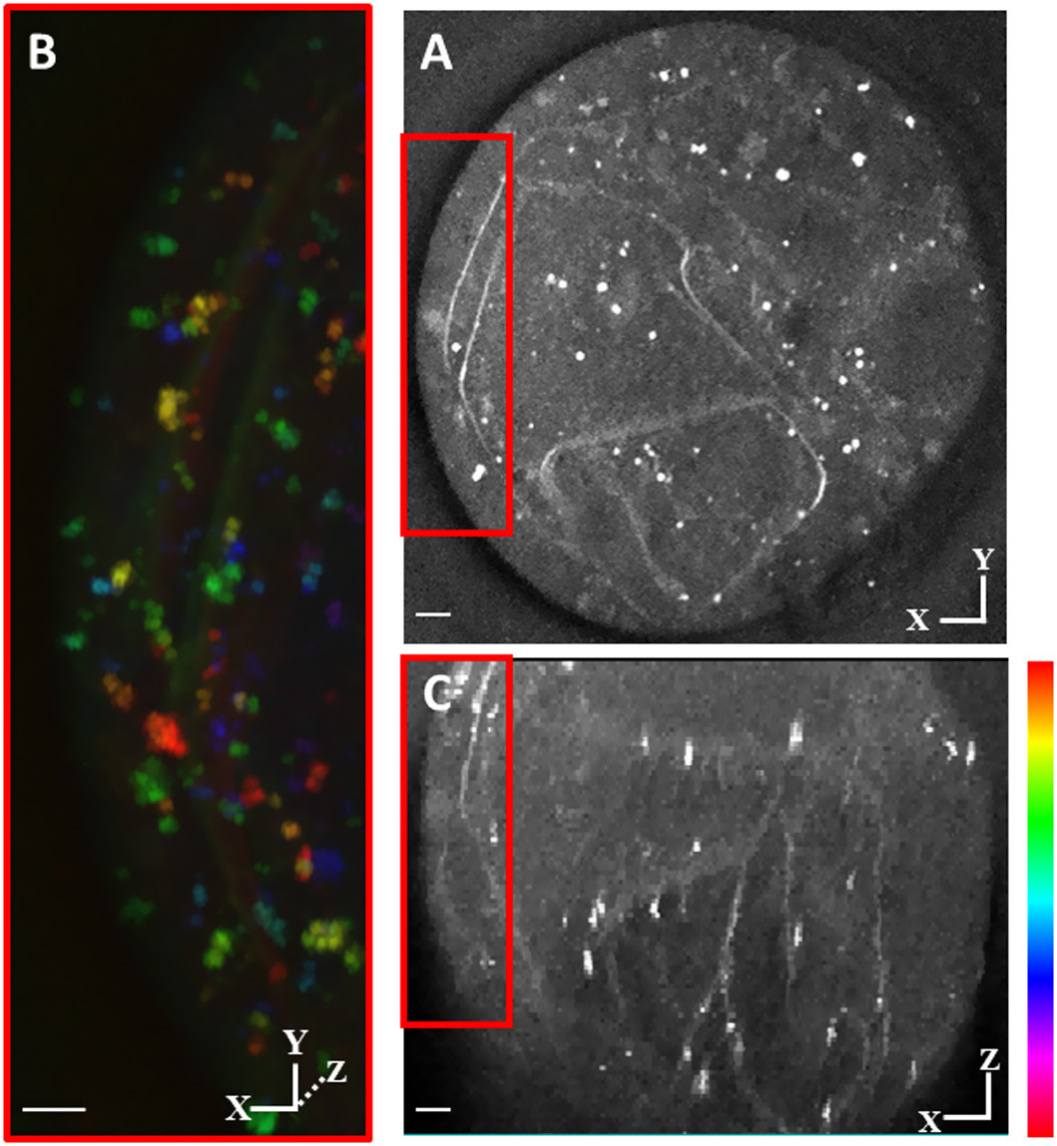
Label-free CARS 3D images in living MII oocytes matured with high PA. For IVM, immature GV stage oocytes were incubated overnight in standard M2 medium supplemented with 200 µM PA. MII oocytes were then selected for CARS imaging. (A) Single z step CARS image (xy section) of the oocyte at wavenumber 2850 cm^−1^. (B) Depth colour-coded images of CARS z-stacks through the same oocytes. (C) The CARS z-stacks of the same oocyte are presented as xz section, CARS was acquired; 0.1 × 0.1 μm xy pixel size; 0.5 μm z-step; 0.01 ms pixel dwell time; ~14 mW (~9 mW) pump (Stokes) power at the sample. Scale bars: 10 μm. Colour bar shows depth colour-coding from −25 to 25 μm of 101 z-stacks (0 μm being the approximately equatorial plane of the oocyte or embryo); the brightness of each colour is the maximum intensity at each corresponding z-plane. Scale bars: 5 μm.

In OA-treated oocytes, or in PA plus OA-treated oocytes (Fig. 6I, J, K, L, M, N, O and P), the sheet structures and the dark holes were not seen. However, some areas of ER tubular matrices become less well-defined on the TPF images (Fig. 6J and N). Such areas have a 3D amorphous shape (Supplementary Fig. 2), and they are distributed throughout the oocytes without any recognizable pattern. Those amorphous structures were also weakly visible in the CARS image at 2850 cm^−1^ (Fig. 6K and O), indicating that they had a high-lipid content. Label-free CARS images (Fig. 7) showed that the ER structures in PA-treated oocytes were not induced by neuro-DiO labelling as it was also visible in CARS images at 2850 cm^−1^ of oocytes not injected with neuro-DiO. The CARS image stacks reveal that those structures had a 3D sheet-like morphology that was distributed across the whole oocytes. These data suggest that PA causes disruption to the ER structure in a way that is not seen with OA, or with co-incubation of OA with PA. These data suggest that PA causes disruption to the ER structure in a way that is not seen with OA, but can be supressed by co-incubation of OA with PA.

Hyperspectral CARS microscopy can acquire a spectrum of CARS frequencies for each spatial point, providing quantitative information on the chemical composition of cellular structures (Masia *et al.* 2013, Bradley *et al.* 2016, Karuna*et al.* 2019). A region (20 μm × 20 μm) in the oocyte that contained both a LD and the sheet structure was chosen for hyperspectral imaging to elucidate their chemical compositions; xy images across the CH-stretch frequencies (2600–3800 cm^−1^, 5 cm^−1^ step) were acquired to obtain vibrational spectra of LDs in different spatial positions in the regions of interest. The CARS third-order susceptibility spectra (Supplementary Fig. 3) showed that the LDs and the sheet structures have the same chemical components with well-characterized vibrational peaks of methine (C-H), which suggests that the sheet structures mainly consist of lipids and that the protein content on/around those structure is likely to be as low as in LDs.

**Figure 8:**
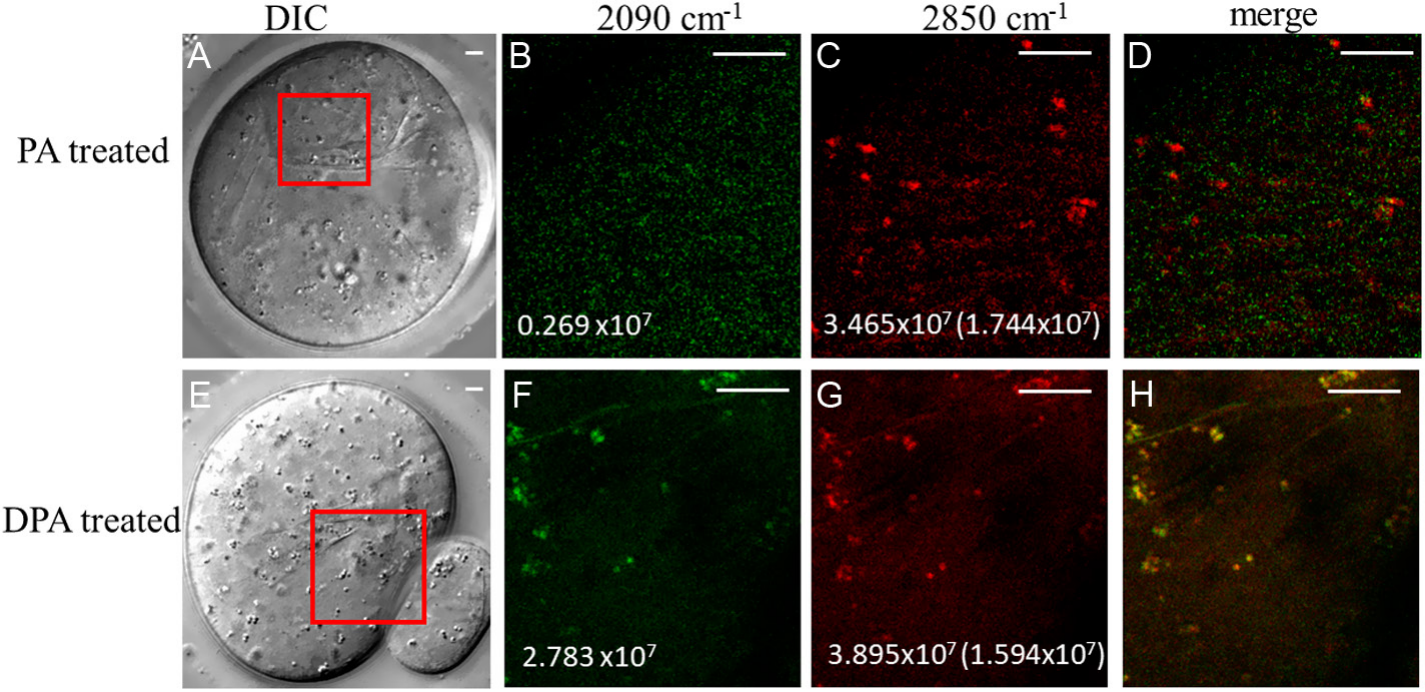
CARS images of living MII oocytes matured in DPA. Immature GV stage oocytes were incubated in standard M2 medium supplemented with 200 µM PA (A, B, C, D) or 200 µM DPA (E, F, G, H) for overnight. MII oocytes were then selected for DIC (A, E) (as in Fig. 3) false-coloured CARS images at wavenumber 2850 cm^−1^ (C, G) and at wavenumber 2090 cm^−1^ (B, F), accompanied by (D, H) false-coloured overlays. Maximum CARS intensities are shown in photoelectrons per second. Values in C and G denote the intensity under excitation conditions identical to those in B and F, respectively, that is they are corrected for the varying temporal overlap of pump and Stokes at different wavenumbers. Scale bars: 5 μm.

We have previously shown that deuterium-labelled OA can be used to image specific FA incorporated into LDs. To establish the causes of the sheet structures, GV oocytes, were matured in 200-μM deuterated palmitic acid (DPA). The incorporation of deuterium in PA allows CARS microscopy to specifically resolve regions of PA uptake. Figure 8 shows that the LDs and sheet structures both have a well-resolved CARS image contrast at 2090 cm^−1^, that is in the region of the Raman spectrum expected for deuterated lipids (see also Supplementary Fig. 3), which is absent from the structures in normal PA-treated oocytes. The LDs and sheet structures in DPA-treated oocytes were visible on both CARS images at 2850 cm^−1^ and 2090 cm^−1^, whereas in PA-treated oocytes they were only visible at 2850 cm^−1^ (Fig. 8). These data show that the lipids in the sheet structures of the ER contained a substantial amount of externally added PA. This result suggested a major effect of PA in mouse oocytes is to partition into the ER membranes and cause a substantial disruption of the morphology of the ER.

## Discussion

In this report, we investigated the effects of FA treatment during IVM on mitochondrial metabolism, Ca^2+^ physiology, LD spatial distribution and ER structure. We have used similar concentrations of PA and OA from previous studies that showed that saturated FAs such as PA can cause ER stress and defects in mitochondrial metabolism. Previous studies on oocytes or somatic cells used concentrations of 400–500 μM (Karaskov *et al.* 2006, Wu *et al.* 2012). The concentration of PA and OA in follicular fluid in some women who had relatively poor *in vitro* fertilization outcomes is around the 100 μM range (Jungheim *et al.* 2011). Hence, the effects we find here at 200-μM PA and OA are therefore within the range used in other studies on ER stress and within the range of physiological relevance. These concentrations were not toxic to oocytes which *in vivo* would be exposed to PA for many weeks, as opposed to less than a day in our *in vitro* studies. We used 15 h for our maturation to maximize exposure, and this duration is similar to that used in previous studies of oocyte maturation (Wu *et al.* 2012), as well as being comparable in time frame to that we study *in vivo* matured super-ovulated oocytes. We used CARS imaging to show that there is a clear decrease in the LD size alongside an increase in LD clustering following IVM treatment with PA. Conversely, incubation with OA results in LD sizes and spatial distributions similar to control MII oocytes. Most significantly we found that PA incubation causes disruption of the ER structure. These change in ER structures occurred in the absence of any consistent changes in mitochondrial activity or Ca^2+^ homeostasis. Our data suggest that the disruption of ER membranes is a primary factor in PA-induced toxicity in oocytes.

We investigated whether FA treatment induces changes in mitochondrial activity because it has been proposed that PA treatment can alter the FAD/FADH redox status, the inner mitochondrial membrane potential and ROS production in mouse oocytes (Igosheva *et al.* 2010, Wu *et al.* 2010, Dunning *et al.* 2014). The data suggest that oocyte mitochondrial membrane potential is reduced when matured in a high-PA environment (Wu *et al.* 2010). However, others have shown that oocytes from mice on high-fat diets have a higher mitochondrial membrane potential (Igosheva *et al.* 2010). It is possible that the different results are due to different methods used to measure mitochondrial membrane potential and it is hence unclear whether a consistent change in mitochondrial potential is seen with excess FA exposure. Oocytes and zygotes isolated from obese mice that were fed with a high-saturated fat and sugar diet have higher FAD autofluorescence intensity than the oocytes from mouse with normal diet (Igosheva *et al.* 2010). This suggests that prolonged periods of high-lipid levels are required to elevate mitochondrial redox status in mature oocytes. Our data showed that high concentrations of PA or OA did not consistently change the FAD/FADH redox state or ATP levels of *in vitro* matured MII oocytes, despite showed changes in LD distribution and morphology. This suggests that changes in mitochondrial metabolism are not the underlying drivers of changes in LDs or in explaining the acute toxic effects of PA in mouse oocytes.

The PA-induced ER stress was shown to significantly reduce the fertilization rate of mouse oocytes (Wu *et al.* 2012). Intracellular Ca^2+^ store homeostasis in mammalian oocytes is crucial for Ca^2+^ signalling during fertilization (Swann & Lai 2013). Disruption of Ca^2+^ homeostasis in the ER can lead to UPR and ER stress (Krebs *et al.* 2011, Groenendyk *et al.* 2013). The expression of the same ER stress markers were increased in mouse oocytes cultured with thapsigargin (Wu *et al.* 2012). However, there is still no direct evidence showing that UPR alters ER Ca^2+^ contents (Krebs *et al.* 2015). It is thought that after prolong ER stress, proapoptotic factors, Bcl2-associated X protein and Bcl-2 homologous antagonist/killer oligomerize to permit Ca^2+^ efflux from ER into the cytoplasm. Increased cytosolic Ca^2+^ can activate both mitochondria-dependent and -independent caspase cascades (Scorrano *et al.* 2003, Malhotra & Kaufman 2007*b*). This Ca^2+^ event should only take place shortly before the cell undergoes programmed cell death. Here we demonstrated that long-term PA or OA exposure during IVM do not alter intracellular Ca^2+^ stores, and addition of PA or OA to immature oocytes did not induce any Ca^2+^ response. Example traces also show that the Ca^2+^ release and recovery have the same dynamics as the control, suggesting that the Ca^2+^ homeostasis is not significantly affected. These results are consistent with previous observations that chronic treatment of PA or OA did not alter the cytosolic Ca^2+^ level in Chinese Hamster Ovary cells (Karaskov *et al.* 2006). Hence, our data are consistent with previous proposals that PA does not alter ER Ca^2+^ homeostasis in surviving cells. Given these observations, it is tempting to speculate that in mouse oocyte, long-term treatment of PA changes the biophysical properties of the ER membrane without significantly altering the intracellular Ca^2+^ or mitochondria functions. ER Ca^2+^ response and mitochondria dysfunction might only take place when cells initiate programmed cell death after being overwhelmed by the prolonged ER stress.

Free FAs are the building blocks for the cellular membrane biogenesis. Both fluorescent staining and label-free, chemically specific CARS imaging showed that PA treatment induced the formation of lipid rich, rigid membrane domains on the ER. These change the normal tubular matrix structure of the ER and potentially can cause membrane dilation. Their morphology resembles PA-induced ER dilation and UPR previously reported using other methods in somatic cells (Karaskov *et al.* 2006, Leamy *et al.* 2014, Shen *et al.* 2017). Biophysical properties of cellular bilayers and monolayers are affected by the lipid composition (Thiam *et al.* 2013). Saturated PLs have a cylindrical shape (have no preferred curvature when forming monolayer) that provides effective coverage of the surface area and lower surface tension, which serve as excellent stabilizing surfactants for the LDs. PLs with unsaturated acyl chains, on the other hand, have predominant hydrophilic part, they are less-optimal surfactants, but important for balancing the rigidity of the membrane as well as generating negative curvature. Whether a pore expands and results in LD fusion depends on the intrinsic curvature of the PL (Kabalnov & Wennerstrom 1996, Bibette *et al.* 2002). Therefore, LDs with saturated PLs in their membranes have hyperstability and are less likely to fuse. This is consistent with our observation that PA-treated oocytes have the greatest numbers of LDs with the smallest size. Co-treatment of PA and OA was able to increase the average LD size, perhaps by LDs fusion, slightly decrease the total LD numbers and result in less LD aggregation. Significant changes in ER and LD membrane fluidity and rigidity can also affect the concentration of LD-associated protein, since they must migrate or insert into the LD formation site on the ER. (McMahon & Gallop 2005, Kory *et al.* 2016). Therefore, high FA treatments affect both the LD distribution and the ER morphology by changing the composition of the PL pool in the oocyte.

The excess FAs taken up by the oocytes are converted into neutral lipids TAG in the inner membrane of ER, where they are packed into LDs. CARS images at 2850 cm^−1^ showed that while the number of LDs was slightly increased, the LD volume and in turn the total LD lipid content were slightly decreased in oocytes matured with high concentration of PA. Conversely, oocytes treated with OA, or a combination of OA and PA showed larger LD sizes, and in turn a total LD content similar to untreated MII oocyte controls. A similar observation was reported in skeletal cell (Coll *et al.* 2008), where supplementary PA was mainly incorporated into DAG in ER membrane whereas supplementary OA was mainly incorporated into TAG in LDs, and co-treatment showed greater incorporation of FA into TAG than cells exposed only to PA. It was proposed that high PA leads to expression of DAG acyltransferase-2 (DGAT2), which is the enzyme that catalyses the conversion of DAG into TAG (Palomer *et al.* 2018). Hence, previous studies in somatic cells have clearly suggested that OA offsets the effect of PA. In our study, the rigid lipid structures on the ER were not found in oocytes being treated with OA or a combination of OA and PA. We propose that, in mouse oocytes, excessive PA cause ER stress by oversaturating the ER membrane and that OA reverses PA-induced ER stress by increasing PA incorporation into TAG in LDs as suggested in somatic cells. Hence, the effects of PA on ER structure and LD formation are interdependent in that promoting LD formation can offset the other such as the ER structure disruption.

The presence of abnormal structures within the ER observed within PA treated oocytes might be the first and clearest indicator of stress within an oocyte. Here, we showed that the abnormal ER structures are originated from excessive PA in the culture environment, which oversaturates and hyperstabilizes and rigidifies parts of the ER membrane after long-term incubation. Our ER staining showed that the abnormal ER structures expanded across the whole eggs, whereas there were no observable changes in the rest of the ER morphology. This may be because some sites of the ER membrane are more prone to excessive incorporation of saturated PLs or accumulation of DAG. The localization of the abnormal ER structures might explain why there were no changes in overall intracellular Ca^2+^ store as the majority of Ca^2+^ channels/pumps on the ER member retain their functions for maintaining Ca^2+^ homeostasis. However, changes in the stability of the overall, or a significant portion of the, ER will affect the ER homeostasis and it can trigger URP. This might help some eggs to survive by adapting to the ER alteration, whereas other eggs are overwhelmed by the prolonged stress response and undergo apoptosis. We also showed that this ER stress is not observed when using unsaturated FA, such as OA, in combination with PA, which suggests that the lipid composition as well as the levels of total FA within the developing follicle can have a significant influence upon oocyte morphology and maturation.

## Supplementary Material

Figure S1. Transient effects of FA and OA on mitochondrial metabolism and intracellular Ca2+ stores of immature oocytes. Example trace of mitochondrial FAD autofluorescence signal in immatures GV stage oocytes after adding 200 µM PA (A) or 200 µM OA (B). The occytes were then subjected to induction of maximum reduction (2 mM cyanide) followed by subsequent maximum oxidation (1 μM FCCP). Data represent multiple eggs (n = 19 or 21 for PA or OA condition respectively), using four mice trials. In part C and D immatures GV stage oocytes were injected with a mixture OGBD and Alexa594 for ratio-metric fluorescent measure of intracellular Ca2+ store. The example trace showed the Ca2+ response of the oocyte after adding 200 µM PA (C) or 200 µM OA (D). For both experiments, thapsigargin (10μM) was then added to induce a transient Ca2+ response. Data represent multiple oocytes (n = 11 or 18 for PA or OA condition respectively), from four independent trials.

Figure S2. CARS and TPF 3D images in MII oocytes. Immatures GV stage oocytes were incubated in standard M2 medium (A) supplemented with 200 µM PA (B), 200 µM OA (C), or combination of 200 µM PA and 200 µM OA (D) for overnight. MII oocytes (eggs) were then selected and injected with Neuro DiI in soybean oil droplet. Z-stack of CARS (red) and TFP (green) images of the eggs were simultaneously acquired and showed as z stack of false-coloured overlays here. In E, F and G, images are shown of a GV stage oocytes incubated in M2 medium supplemented with 200 µM PA and injected with Neuro DiI in soybean oil droplet. There is a Z-stack of the DIC image (E) and TFP fluorescence images of the eggs (in F) also shown as a z stack.

Figure S3. Hyperspectral imaging of chemical content in PA treated oocytes. Spatial and spectral outputs from unsupervised FSC3 analysis of hyperspectral CARS datasets for oocytes matured in 200 µM PA. The concentration maps (A) identify and spatially resolve six components which are chemically specified by their representative spectra showing the phase-retrieved imaginary part of the normalised CARS susceptibility ℑ(□̅ ). Component 3 is identified corresponding to fatty acid since it has the characteristic peak at 2850 cm-1 (B). Volume concentration ranges are shown on a grayscale. DIC images show whole cells and the lipid-rich regions selected for hyperspectral CARS acquisition. Scale bars show 20μm in DIC images and 4μm in FSC3 images (except for lowest row which shows 2μm). (C) and (D) are CARS hyperspectral images of oocytes incubated in DPA (deuterated palmitic acid) overnight. Mature oocytes (eggs) were then selected for DIC and CARS images at wavenumber 2850 cm−1 and at wavenumber 2090 cm−1 (C). Hyperspectral CARS images of the selected area (red outline) were acquired and analysed by HIA (details in the method section; and the spectra of the areas contain the LD (red) and sheet-structure (blue) are shown in (D).

## Declaration of interest

Karl Swann is on the editorial board of Reproduction. Karl Swann was not involved in the review or editorial process for this paper, on which he is listed as an author.The other authors have nothing to disclose.

## Funding

This work and Y W were supported by a UK BBSRC Research Council responsive mode research grant (grant no. BB/P007511/1). P B acknowledges the UK EPSRC Research Council for her Leadership fellowship award (grant no. EP/I005072/1) and the Royal Society for her Wolfson Research Merit Award (grant no. WM140077). I P is funded by the UK EPSRC (grant no. EP/L001470/1) and UK BBSRC (grant no. BB/P007511/1). W L acknowledges support by a Leverhulme Royal Society Research Fellowship (grant No. LT20085). The CARS microscope set up was funded by the UK BBSRC (grant BB/H006575/1 and BB/P007511/1).

## Author contribution statement

Y W, I P, H B-C and E P performed experiments and analysed the data. K S, P B and W L conceived the study. Y W, K S, P B and W L co-wrote the manuscript.
