# Supplementary material for: A primary effect of palmitic acid on mouse oocytes is the disruption of the structure of the endoplasmic reticulum
Source: Reproduction. 2021 Dec 3;163(1):45–56. doi: 10.1530/REP-21-0332 (PMC8801006; doi:10.1530/REP-21-0332)
Supplement: Figure S1. Transient effects of FA and OA on mitochondrial metabolism and intracellular Ca2+ stores of immature oocytes. Example trace of mitochondrial FAD autofluorescence signal in immatures GV stage oocytes after adding 200 µM PA (A) or 200 µM OA (B). The occytes were then subjected to induction  [file supplementary_figure_1.pdf]

Supplementary Figure 1.

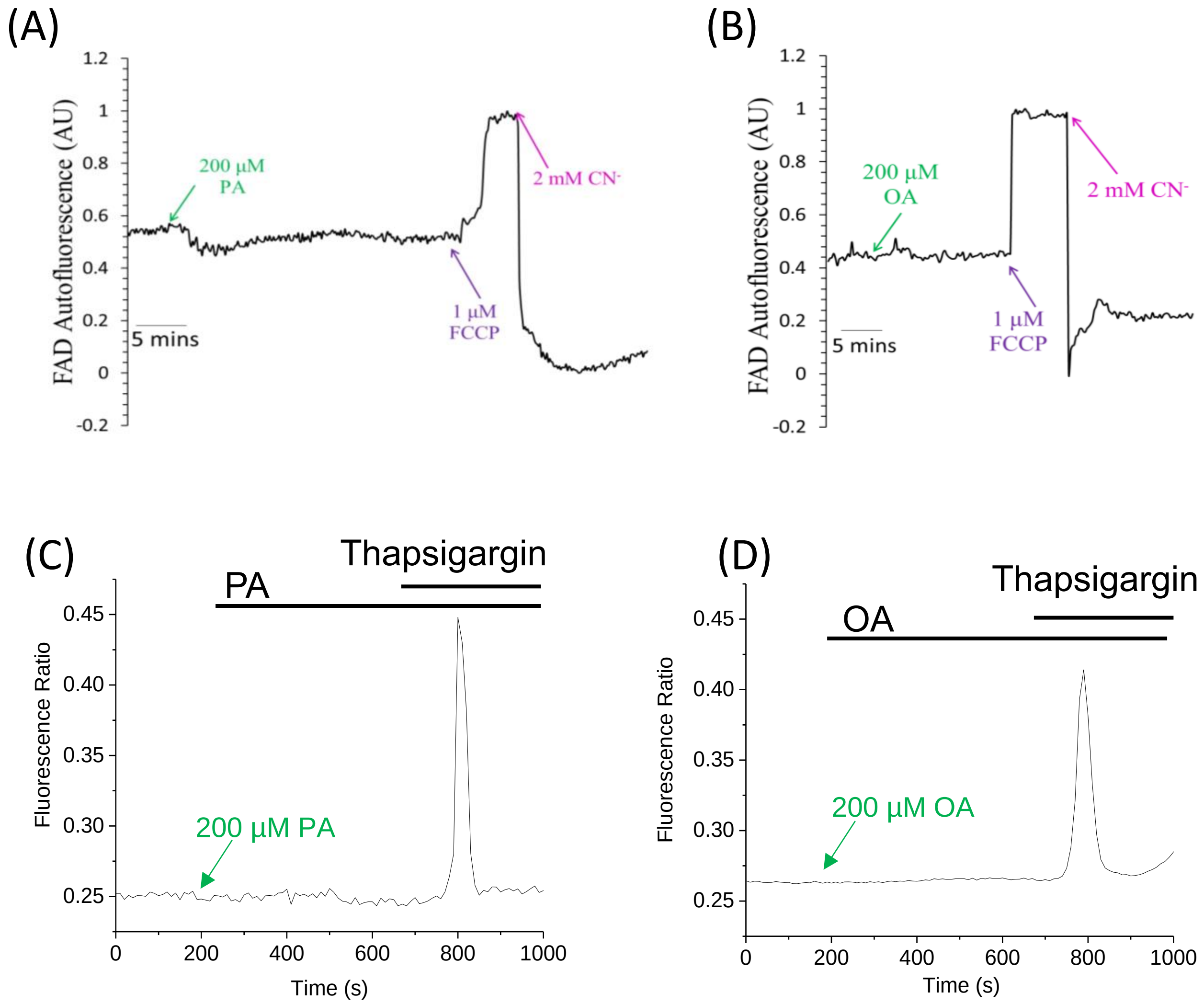

Figure S1. Transient effects of FA and OA on mitochondrial metabolism and intracellular Ca^{2+} stores of immature oocytes.

Example trace of mitochondrial FAD autofluorescence signal in immatures GV stage oocytes after adding 200 µM PA (A) or 200 µM OA (B). The occytes were then subjected to induction of maximum reduction (2 mM cyanide) followed by subsequent maximum oxidation (1 µM FCCP). Data represent multiple eggs (n = 19 or 21 for PA or OA condition respectively), using four mice trials. In part C and D immatures GV stage oocytes were injected with a mixture OGBD and Alexa594 for ratio-metric fluorescent measure of intracellular Ca^{2+} store. The example trace showed the Ca^{2+} response of the oocyte after adding 200 µM PA (C) or 200 µM OA (D). For both experiments, thapsigargin (10µM) was then added to induce a transient Ca^{2+} response. Data represent multiple oocytes (n = 11 or 18 for PA or OA condition respectively), from four independent trials.
