# Supplementary material for: A primary effect of palmitic acid on mouse oocytes is the disruption of the structure of the endoplasmic reticulum
Source: Reproduction. 2021 Dec 3;163(1):45–56. doi: 10.1530/REP-21-0332 (PMC8801006; doi:10.1530/REP-21-0332)
Supplement: Figure S2. CARS and TPF 3D images in MII oocytes. Immatures GV stage oocytes were incubated in standard M2 medium (A) supplemented with 200 µM PA (B), 200 µM OA (C), or combination of 200 µM PA and 200 µM OA (D) for overnight. MII oocytes (eggs) were then selected and injected with Neuro DiI in soyb [file supplementary_figure_2.pdf]

Supplementary Figure 2.

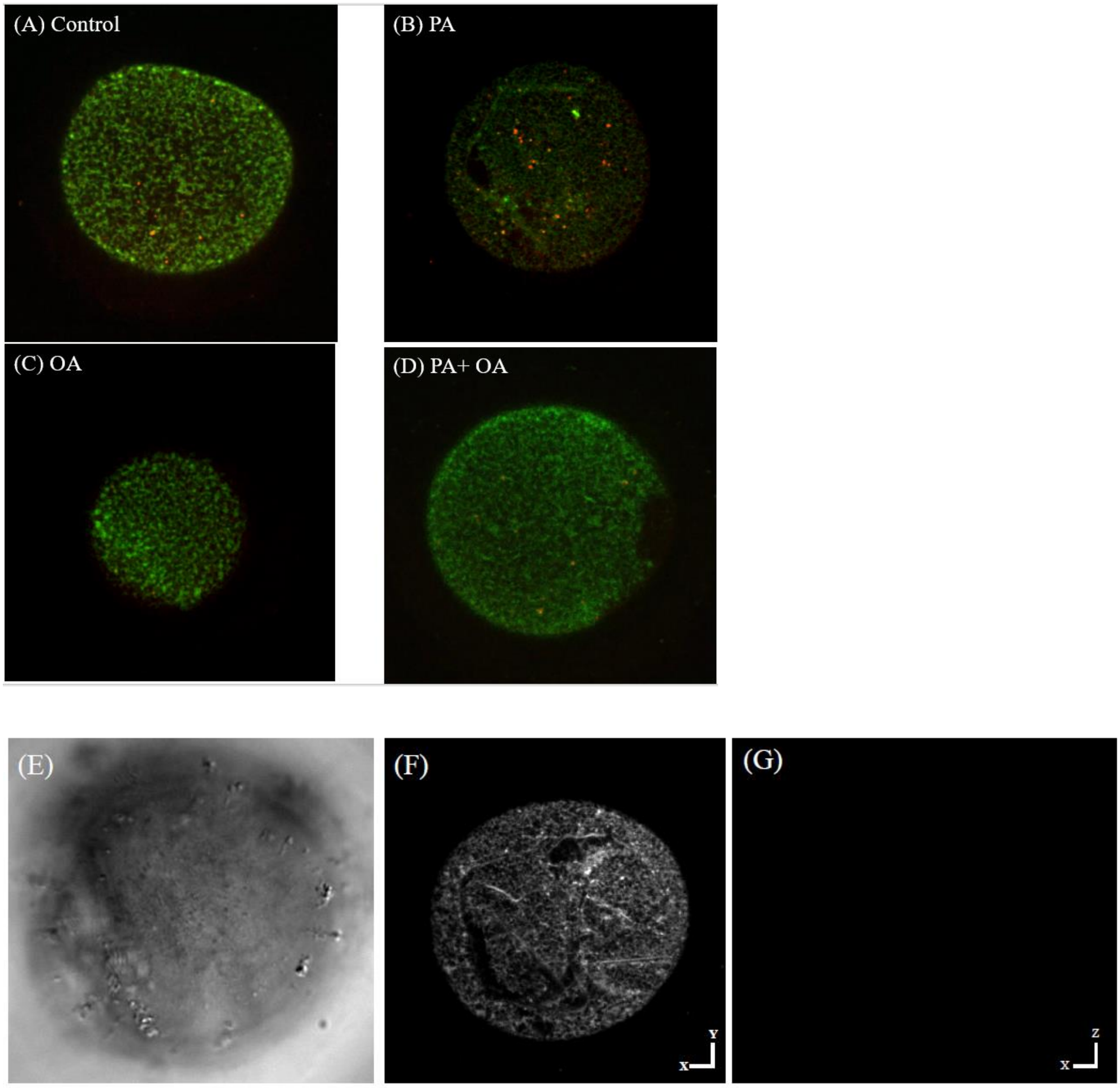

Figure S2. CARS and TPF 3D images in MII oocytes.
Immatures GV stage oocytes were incubated in standard M2 medium (A) supplemented with 200 µM PA (B), 200 µM OA (C), or combination of 200 µM PA and 200 µM OA (D) for overnight. MII oocytes (eggs) were then selected and injected with Neuro DiI in soybean oil droplet. Z-stack of CARS (red) and TFP (green) images of the eggs were simultaneously acquired and showed as z stack of false-coloured overlays here. In E, F and G, images are shown of a GV stage oocytes incubated in M2 medium supplemented with 200 µM PA and injected with Neuro DiI in soybean oil droplet. There is a Z-stack of the DIC image (E) and TFP fluorescence images of the eggs (in F) also shown as a z stack.
