# Supplementary material for: A primary effect of palmitic acid on mouse oocytes is the disruption of the structure of the endoplasmic reticulum
Source: Reproduction. 2021 Dec 3;163(1):45–56. doi: 10.1530/REP-21-0332 (PMC8801006; doi:10.1530/REP-21-0332)
Supplement: Figure S3. Hyperspectral imaging of chemical content in PA treated oocytes. Spatial and spectral outputs from unsupervised FSC3 analysis of hyperspectral CARS datasets for oocytes matured in 200 µM PA. The concentration maps (A) identify and spatially resolve six components which are chemically spec [file supplementary_figure_3.pdf]

Supplementary Figure 3.

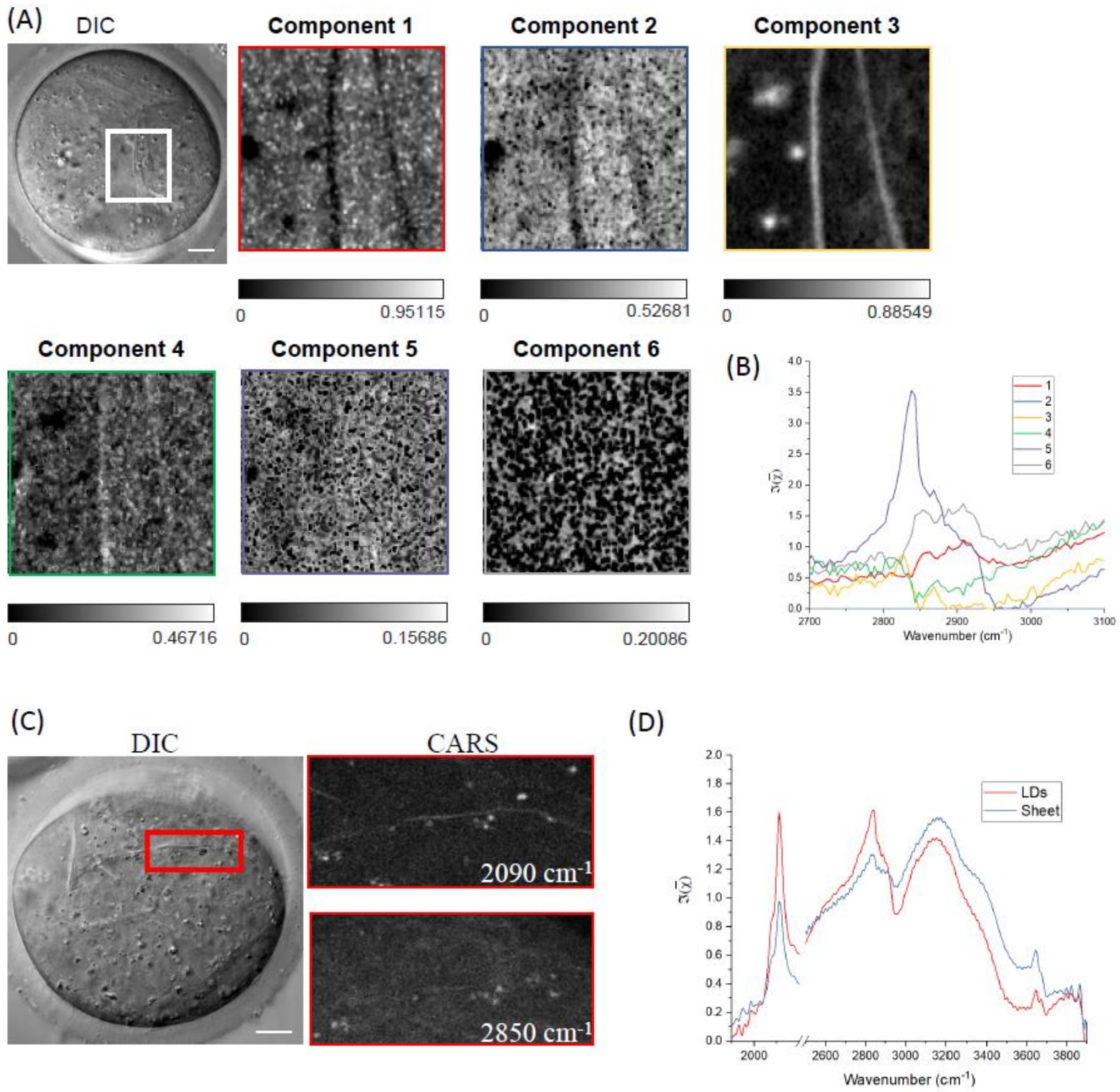

Figure S3. Hyperspectral imaging of chemical content in PA treated oocytes.
Spatial and spectral outputs from unsupervised FSC^3 analysis of hyperspectral CARS datasets for oocytes matured in 200 µM PA. The concentration maps (A) identify and spatially resolve six components which are chemically specified by their representative spectra showing the phase-retrieved imaginary part of the normalised CARS susceptibility ℑ(□̄). Component 3 is identified corresponding to fatty acid since it has the characteristic peak at 2850 cm^{-1} (B). Volume concentration ranges are shown on a grayscale. DIC images show whole cells and the lipid-rich regions selected for hyperspectral CARS acquisition. Scale bars show 20µm in DIC images and 4µm in FSC3 images (except for lowest row which shows 2µm). (C) and (D) are CARS hyperspectral images of oocytes incubated in DPA (deuterated palmitic acid) overnight. Mature oocytes (eggs) were then selected for DIC and CARS images at wavenumber 2850 cm^{-1} and at wavenumber 2090 cm^{-1} (C). Hyperspectral CARS images of the selected area (red outline) were acquired and analysed by HIA (details in the method section; and the spectra of the areas contain the LD (red) and sheet-structure (blue) are shown in (D).
